# Homosexuallity in the eastern soceity

**DOI:** 10.1192/j.eurpsy.2021.2156

**Published:** 2021-08-13

**Authors:** V. Mahasneh

**Affiliations:** Psychiatry, Private Clinic, Damascus, Syria

## Abstract

**Introduction:**

Everybody Knows the murmurs about homosexuallity that make harm to those humans especially in the Eastern Society.

**Objectives:**

We (as Mental Health Professionals) should struggle against Stigma of Homosexuality as well as psychoeducate others about their human rights.

**Methods:**

As a psychiatrist as well as EMDR Clinician Practitioner, i interviewed and still interview many clients who are homosexual. The first sessions, they are afraid to talk about their situation because of the Stigma as well as i am a Muslim, and their bad expeirence when they went to some psychiatrists who tortured them verbally. Withregard many of them tell me (i pray, fast, etc.), but their families and religious leaders say to them (you are not faithful) as judgement for them. Even me, i was attacked by some others (for example : Religious Leaders …) because i say homosexuality is normal.

**Results:**

Homosexualls till now are tortured (Verbally, Physically and Sexually) in general, and especially in the Eastern Sosciety.

**Conclusions:**

We should work more and more to psychoeducate others about homosexuallity especially the religious leadrers that those are humans and we should respect their human rights. And this Stigma should be DELETED from the MIND.

**Disclosure:**

No significant relationships.

